# No chikungunya virus infections among Dutch long-term travellers to (sub)tropical countries: a prospective study 2008–2011

**DOI:** 10.1186/s12879-019-3819-4

**Published:** 2019-02-26

**Authors:** Femke W. Overbosch, Floor Elfrink, Janke Schinkel, Gerard J. B. Sonder

**Affiliations:** 1 Department of Infectious Diseases, Public Health Service (GGD), Amsterdam, The Netherlands; 2 National Coordination Centre for Traveller’s Health Advice (LCR), Amsterdam, The Netherlands; 3 Department of Medical Microbiology, Laboratory of Clinical Virology, Academic Medical Center, Amsterdam, the Netherlands; 4 Department of Internal Medicine, Division of Infectious Diseases, Tropical Medicine and AIDS, Academic Medical Center, Amsterdam, The Netherlands

**Keywords:** Chikungunya, Travellers, Prospective

## Abstract

**Background:**

Chikungunya is an arthropod-borne viral disease now identified in over 60 countries in Asia, Africa, Europe, and the Americas. Chikungunya virus (CHIKV) has spread in the last 15 years to many countries, causing large local outbreaks. CHIKV infection can be clinically misdiagnosed in areas where dengue and/or Zika infections occur. Prospective studies are necessary to calculate the true incidence rate of CHIKV infection in travellers. The aim of this study was to obtain the attack and incidence rates of CHIKV infection among long-term travellers and identify associated risk factors.

**Methods:**

A previously collected prospective cohort of Dutch long-term travellers (12–52 weeks) to subtropical and tropical countries was tested. From December 2008 to September 2011, participants were recruited at the travel clinic of the Public Health Service Amsterdam. A weekly diary was kept during travel in which participants recorded their itinerary, symptoms, and physician visits. On return, their pre- and post-travel blood samples were tested for the presence of IgG antibodies to CHIKV antigen. Seroconversions were confirmed by an in-house CHIKV neutralisation test.

**Results:**

The median age of 603 participants was 25 years (interquartile range [IQR]: 23–29); 35.7% were male; median travel duration was 20 weeks (IQR: 15–25), and purpose of travel was predominantly tourism (62%).

The presence of anti-CHIKV IgG in the pre-travel sample, suggestive of previous CHIKV infection, was found for 3/603 participants (0.5%); all three had been previously travelling in either Africa or Asia. In one traveler who visited Latin America, a seroconversion was found (0.2%) but the CHIKV neutralisation test was negative, making the incidence rate 0.

**Conclusion:**

No chikungunya virus infections were found in this 2008–2011 prospective cohort of long-term travellers. We recommend the research be repeated, particularly as the sample size of our cohort might have been too small. Also, extensive spread of chikungunya virus has likely increased incidence rates among travellers since 2013.

## Background

Chikungunya is a mosquito-borne viral disease whose agent belongs to the alphavirus genus of the family Togaviridae, and the mosquitoes most commonly involved as vector are the daytime-active *Aedes aegypti* and *Aedes albopictus*. Symptoms of CHIKV infection can be mild and unrecognized or confused with the similar symptoms of dengue and Zika virus infection [1–3]. However, chikungunya’s characteristic symptoms are high fever accompanied by severe arthralgia, which can be debilitating [1]. There is no specific treatment for chikungunya, and no vaccine is available [1].

The first recorded chikungunya epidemic was reported in Tanzania in 1952. The disease was subsequently reported in other parts of Africa, Asia, and the Indian subcontinent [1]. In 2007, the first local transmission was reported in Europe, and after the first autochthonous case was reported at the Caribbean island of St Martin in 2013, over a million cases were reported in the Americas within 9 months [1, 4].

Due to the emerging CHIKV endemicity and recent reports of CHIKV infections among travellers in endemic countries [3, 5–13], we were interested in the CHIKV incidence rate in Dutch travellers. Therefore, we tested a previously collected prospective cohort of Dutch long-term travellers (12–52 weeks) to subtropical and tropical regions [14]. Our primary aim was to estimate the attack rate and incidence rate of CHIKV infections during travel in Africa and Asia and to identify the associated risk factors. As we were also curious as to possible exposure in countries with no evidence of autochthonous CHIKV transmission at the time of our study, we also tested samples of all cohort travellers to Latin America [15, 16].

## Methods

### Study population and study procedure

The study design and sample collection methods of the long-term travellers study have been described in detail previously [14]. In brief, this study was conducted as part of a prospective mono-centre study of long-term travellers aged ≥18 years to subtropical and tropical countries who were recruited at the Public Health Service travel clinic in Amsterdam from December 2008 through September 2011. Long-term travel was defined as travel for ≥12 and ≤ 52 weeks.

A standardised questionnaire in Dutch or English was used to collect data before departure on individual socio-demographics, travel history, and purpose of travel. Travellers were asked to keep a structured, weekly travel diary. Travellers gave a blood sample once during their pre-travel visit and once 2–6 weeks after return.

### Laboratory methods

After all study participants had returned, all post-travel serum samples were tested for immunoglobulin (Ig) G antibodies to CHIKV antigen by using an anti-CHIKV enzyme-linked immonosorbent assay (ELISA) IgG test (Euroimmun, Lübeck, Germany), according to manufacturer’s instructions. For participants whose post-travel sample yielded a positive test result for anti-CHIKV IgG, their pre-travel sample was also tested for anti-CHIKV IgG.

Travel-acquired infection was considered the primary interest of this study. The presence of anti-CHIKV IgG in both the pre- and post-travel sample was considered suggestive of a previous CHIKV infection. Participants with a previous CHIKV infection were considered no longer at risk for a travel-acquired CHIKV infection. The presence of anti-CHIKV IgG in the post-travel sample, together with the absence of anti-CHIKV IgG test in the pre-travel sample, was considered a seroconversion. It was considered as evidence for a CHIKV infection if confirmed by a positive in-house CHIKV neutralisation test (Erasmus University Medical Center, Rotterdam, the Netherlands).

### Statistical analysis

Analysis of destinations was performed at continent-level.The use of DEET (N, N-diethyl-meta-toluamide) was quantified by dividing the number of reported weeks of DEET usage, by the total number of travel-weeks.

Possible symptoms of a travel-acquired CHIKV infection were described using data reported in the travel diaries. Fever with arthralgia in ≥2 joints were considered the most characteristic symptoms of CHIKV infection. Myalgia, headache, skin rash, or vomiting reported simultaneously with fever and arthralgia in ≥2 joints were considered as frequently accompanying symptoms of CHIKV infection. The combination of fever and arthralgia in ≥2 joints followed by symptoms of arthralgia in ≥2 joints in consecutive week(s) was considered suggestive of persisting arthralgia after CHIKV infection.

To identify potential risk factors, following variables were selected to study: sex, age, purpose of travel, visited continent, and use of DEET. The prevalence of previous CHIKV infection and the corresponding 95% confidence interval (CI) were calculated. A *p*-value < 0.05 was considered significant (STATA).

## Results

### Characteristics of the study population

The prospective cohort consisted of 603 participants which formed the study population. The median age was 25 years (interquartile range [IQR]: 23–29), 35.7% were male, and the median interval between return from travel and post-travel blood donation was 25 days (IQR 21–33).

Results suggestive of previous CHIKV infection were found in both pre- and post-travel samples for 3/603 participants (0.5%; 95% CI -0.066-1.1%) (Table 1). All three had been either in Africa or Asia before.

**Table 1:** Characteristics of 603 Dutch long-term travellers including the prevalence of previous chikungunya virus infection

			Previous CHIKV*	
Characteristic	Total, no.	%	No.	%
No. participants	603	100	3	0.5
Sex				
Female	388	64	2	0.5
Male	215	36	1	0.5
Median age, years (IQR†)	25 (23–30)			
Age, years				
< 24	203	34	1	0.5
24–29	261	43	0	0
≥ 30	139	23	2	1.4
Total duration of(sub)tropical travel prior to study, in months				
< 1	263	44	1	0.4
1–3	116	19	1	0.9
> 3	224	37	1	0.5

The participants attended and were recruited at a Dutch travel health clinic between December 2008-September 2011

*CHIKV chikungunya virus, †*IQR* interquartile range

### Travel-acquired CHIKV infection

The median travel duration was 20 weeks (IQR: 15–25); purpose of travel was predominantly tourism (62%), and the three most-visited countries were Thailand (175/600), Indonesia (137/600) and Argentina (130/600) (Table 2). Only one CHIKV seroconversion was found in the 600 participants at risk for CHIKV infection. This participant had travelled in 2011 for 7.5 months through Argentina, Bolivia, Chile, and Peru, and reported no fever nor physical symptoms except coughing for three consecutive weeks. Moreover, the CHIKV neutralisation test was negative. Therefore we found no evidence of travel-acquired CHIKV infection in this cohort of travellers.

**Table 2:** Travel-related characteristics of 600 Dutch long-term travellers at risk for CHIKV infection

Characteristic	Total, no.	%
No. participants	600	100
Median duration of travel, weeks (IQR)	20 (15–25)	
Duration of travel, weeks		
< 16	167	28
16–20	156	26
21–25	146	24
≥ 26	131	22
Purpose of travel		
Tourism	371	62
Work/education	173	29
VFR^^^/other	56	9
Visited continents		
Asia	269	45
Africa	107	18
Latin America	224	37
Use of DEET*, % of total travel duration		
< 25	175	29
25–50	134	22
51–75	102	17
≥ 75	189	32

The participants attended and were recruited at a Dutch travel health clinic between December 2008–September 2011

†*IQR* interquartile range, ^^^*VFR* visiting friends & relatives, *DEET N,N-diethyl-meta-toluamide

The characteristic symptoms of possible chikungunya (fever and in ≥2 joints) were reported by 40/600 (6.7%) participants. Frequently accompanying symptoms were: headache (85%, 34/40), myalgia (90%, 36/40), skin rash (23%, 9/40) and/or vomiting (38%, 15/40).

One of these 40 participants was diagnosed with chikungunya during travel whilst having joint pain and fever. This participant was also the only one who persisted in reporting pain in ≥2 joints in the 12 following weeks until the study ended. The participant had travelled predominantly in India in 2010, but the travel diary did not include information on how the diagnosis was made. Seroconversion for CHIKV was not found in this traveller.

## Discussion

The results of this 2008–2011 study of long-term travellers indicate a negligible risk for Dutch travellers to contract a CHIKV infection, since none of the 600 at-risk participants seroconverted. The results are in line with the available data that CHIKV was not yet introduced in the Americas at the time of the study period. The lack of seroconversion in Asia and Africa was rather unexpected, however, as 40/600 participants reported symptoms which could be characteristic of CHIKV infection. Large outbreaks of chikungunya were described in Asia preceding the study period [1, 17]. During the study period, the virus continued to spread in Southeast Asia, where large outbreaks were reported from popular tourist destinations in Indonesia and Thailand [18, 19]. As a substantial number of our cohort visited these two countries, exposure to CHIKV would have been likely. Concurrent to our study, the EuroTravNet study, investigated the proportion of chikungunya and indeed found some CHIKV infections (0.2% of 6957 and 0.4% of 7408 febrile returning travellers in 2008 and 2010, respectively); however it confirmed that the proportion of travellers with chikungunya was substantially lower than the proportion with dengue. In 2010; 357 of 7408 persons (5%) contracted dengue [7].

Misdiagnosis seemed likely in our one participant who reported characteristic symptoms and chikungunya diagnosis during travel, but showed no CHIKV seroconversion in the post-travel sample. Surprisingly, this participant was not one of the travellers who seroconverted for dengue virus [14], and thus another pathogen probably caused all the symptoms.

Since we found no seroconversions, we could not calculate incidence rate ratios nor perform regression analysis to identify possible risk factors for travel-acquired CHIKV infection. Mosquito-borne infections depend often on seasonality including the wet seasons, as higher temperatures and heavy rainfall influences breeding sites. Possible explanations of our finding no CHIKV infection could be that travellers avoided wet-season-related outbreak areas or that anti-mosquito measures, like fumigation or spraying of insectides, were more extensively implemented in tourist areas than elsewhere. On the other hand, as 39 travellers of our cohort (6.5%) contracted dengue, local anti-mosquito measures cannot be the only reason why no one contracted chikungunya [14].

Our study has some limitations. First, selection bias may have occurred, as all participants were seeking pre-travel health advice when recruited and thus perhaps had a higher health awareness. Second, we did not collect information about specific areas that participants visited in the countries we studied. Therefore, we do not know if participants specifically avoided wet-season-related outbreak areas which might have influenced our incidence findings. Third, the diagnostic test we used probably does not have a 100% sensitivity and could therefore underestimate the true incidence. Fourth, self-reported diaries introduce some bias, though they might be more accurate than recall influenced post-travel questionnaires. Finally, to our knowledge, prospective estimates of the incidence of chikungunya among travellers have not been published before. Probably, the incidence of chikungunya was much lower than for dengue at the time of our study. Therefore, the sample size of our prospective cohort might have been too small to reflect a solid incidence.

## Conclusion

No CHIKV infections were found in this 2008–2011 prospective study among long-term Dutch travellers. Due to the extensive spread of the virus in the Americas since 2013, incidence rates among travellers have likely increased. We therefore recommend the study be repeated, preferably in a larger cohort of travellers. Travellers should be well informed about emerging arthropod-borne infectious diseases and urged to take appropriate anti-mosquito measures.

## Abbreviations

CHIKV: chikungunya virus
CI: confidence interval
DEET: N,N-diethyl-meta-toluamide
ELISA: enzyme-linked immunosorbent assay
IgG: Immunoglobulin G
IQR: interquartile range
